# Polysplenia Syndrome Associated With Heterotaxy of Abdominal Viscera and Type IV Intestinal Atresias 

**Published:** 2012-07-01

**Authors:** Safwan Ahmad

**Affiliations:** Department of Pediatric Surgery, The Children’s Hospital and the Institute of Child Health, Lahore, Pakistan.

**Dear Sir**

Polysplenia syndrome in association with situs inversus and intestinal atresia is rarely reported [1]. A 2-day-old male neonate was presented with neonatal intestinal obstruction. Abdominal radiograph showed air fluid levels suggestive of small bowel atresia. Ultrasound of the abdomen showed dilated bowel loops throughout the abdominal cavity. At operation, there was right sided stomach, centrally located liver, three spleens at normal location, and type IV small bowel atresias (Fig. 1,2). All the small bowel atresias were resected and end to end jejuno-ileal anastomosis was performed. One of the spleens which had a narrow pedicle was removed to prevent any future torsion. The postoperative course remained stormy and patient succumbed to sepsis on 5th postoperative day.

**Figure:**
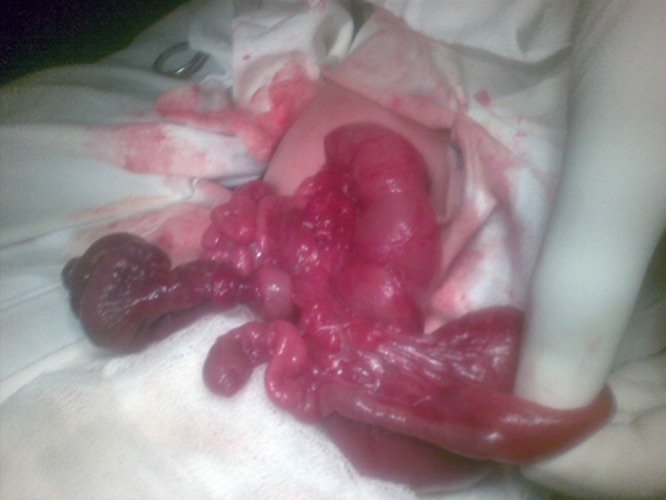
Figure 1: Multiple small bowel atresias- Type IV.

**Figure:**
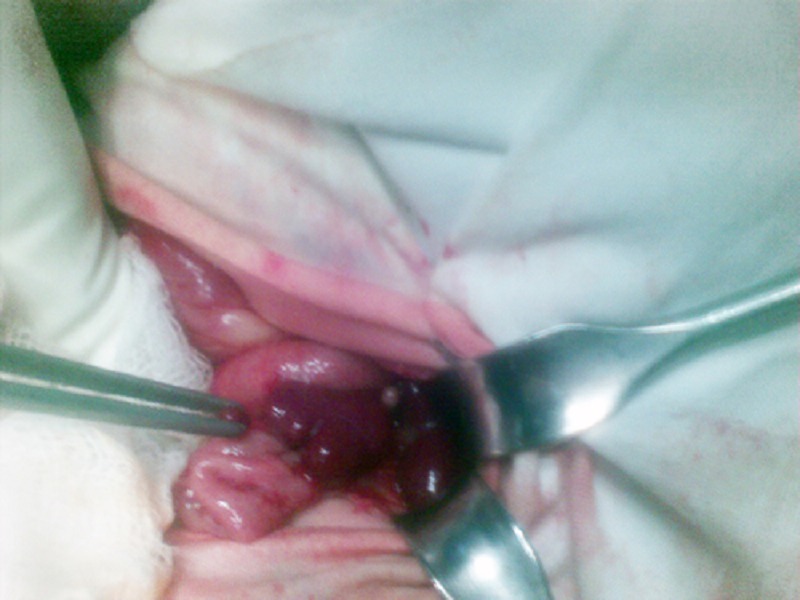
Figure 2: Polysplenia at normal location.

Polysplenia syndrome is associated with numerous anomalies [1-4]. In 20% of cases, heterotaxy of abdominal or thoracic viscera is present. Extreme form of heterotaxy such as situs inversus is not always present [2,3]. Similarly, in our case the stomach was right sided (mirrored), however, the spleens were present at their usual location. Children with this defect usually do not reach to their school going age. Very few cases of polysplenia syndrome are associated with intestinal atresias. Polysplenia syndrome has been associated with duodenal and jejunal atresias [1,4]; its association with multiple atresias is however not reported before.

## Footnotes

**Source of Support:** Nil

**Conflict of Interest:** None

